# Editorial: Artificial Intelligence in Geriatric Mental Health Research and Clinical Care

**DOI:** 10.3389/fpsyt.2022.859175

**Published:** 2022-03-01

**Authors:** Helmet T. Karim, Ipsit V. Vahia, Andrea Iaboni, Ellen E. Lee

**Affiliations:** ^1^Department of Psychiatry, University of Pittsburgh, Pittsburgh, PA, United States; ^2^Department of Bioengineering, University of Pittsburgh, Pittsburgh, PA, United States; ^3^Division of Geriatrics, McLean Hospital, Belmont, MA, United States; ^4^Department of Psychiatry, Harvard Medical School, Boston, MA, United States; ^5^Knowledge, Innovation, Talent, Everywhere (KITE), Toronto Rehab Institute, University Health Network, Toronto, ON, Canada; ^6^Department of Psychiatry, University of Toronto, Toronto, ON, Canada; ^7^Department of Psychiatry, University of California, San Diego, San Diego, CA, United States; ^8^Sam and Rose Stein Institute for Research on Aging, University of California, San Diego, San Diego, CA, United States; ^9^Desert-Pacific Mental Illness Research Education and Clinical Center, Veterans Affairs San Diego Healthcare System, San Diego, CA, United States

**Keywords:** artificial intelligence, older adults, mental health, machine learning (ML), natural language processing (NLP)

Though adoption of artificial intelligence (AI) has been delayed in mental health research and clinical care relative to other fields, AI could potentially enhance diagnostic, prognostic, and treatment approaches for the growing aging population. With ubiquitous usage of wearable sensors, advancements in explainable AI, and growing acceptance of AI in medicine, these approaches could support increasing clinical demands. Despite enthusiasm for AI, usage in clinical settings is tempered by validity and ethical concerns. Integrating AI in clinical settings will require collaborations between clinicians and AI experts, inclusive study samples, and rigorous evaluation (akin to clinical trials for pharmacotherapies).

This special issue is a platform to highlight new AI applications in geriatric mental health research and care and bridge clinical with AI expertise by describing conceptual and pragmatic approaches. This issue showcases varied AI approaches [machine learning, natural language processing (NLP)] applied to multiple data-streams (sensors, electronic health records, interview data, neuroimaging) from multidisciplinary international perspectives. The included papers think broadly about policy and systemic implications and respect ethical concerns and patient protections.

Two manuscripts demonstrate utility in identifying important endpoints and optimizing treatments. Grzenda et al. used two studies to predict treatment outcome in late-life depression using structural imaging. Incorporation of structural imaging improved prediction when combined with clinical markers to two different treatments (antidepressants vs. Tai Chi), indicating that structural markers improve detection of treatment resistance—which allows for more aggressive treatments earlier. Chowdhury et al. conducted a systematic review of the use of electronic health records for predicting various outcomes (primarily dementia). Despite reporting high heterogeneity in data utilized, approaches used to standardize data, and even modeling approaches, the authors acknowledged rapid growth—with 21 studies in the past 5 years. Overall, EHR-integrated AI has potential to aid clinicians—triggering cognitive screening of patients at “high-risk” for dementia or suggesting more proactive approaches for patients at “high-risk” for treatment-resistance. AI models must incorporate clinician feedback and treatment outcomes and undergo dynamic updating. These models should identify which features most strongly predict “high-risk”, thus improving clinician trust and condensing and compiling complex information into a comprehensible report.

Three manuscripts focused on NLP, i.e., deciphering unstructured text to provide insights into social functioning and depression. Badal et al. identified linguistic features (first-person plural pronouns) from interviews about social relationships that predicted scores on social support and loneliness and elicited gender differences. Yamada et al. identified acoustic, prosodic, and linguistic features (inflections, pauses, second formant frequencies, filler and positive words) from interviews on daily life and functioning that were associated with higher loneliness. DeSouza et al. provide an overview of NLP in late-life depression, including tool development for real-time analysis and usage in non-clinical settings, utility as a diagnostic tool, and key ethical/legal concerns and comfort with technologies. Speech data has particular relevance as a primary evaluative technique in psychiatry. However, further work is needed to combine speech with other clinical data to refine our predictive models (e.g., longitudinal data due to individual- and language/dialect-specific issues), to navigate privacy concerns when recording speech patterns, and examine novel data sources (e.g., social media posts and videos).

Three papers analyze passive sensor data to infer how older adults live their daily lives. Recently, around 60% of seniors in North America owned smartphones (1, 2), which will continue to increase. Smart home sensor technologies that monitor living environments are increasing in acceptability and use (1). The main challenge is uncovering meaningful and actionable information within complex data. Lee et al. described using smartphone data (e.g., number of unlocks, time spent at home), that can be used to evaluate behavior and mood over time in older depressed adults receiving psychotherapy. Zulueta et al. examine relationships between keystroke dynamics and cognitive function in people at risk of bipolar disorder. Zhang et al. demonstrate use of an environmental radio sensor to monitor breathing and behaviors of older adults with COVID-19. These were associated with measures of health, cognitive function, and wellbeing. A common challenge was addressing heterogeneity of behaviors and their context where clinical interpretation was not always straightforward. For example, increased behavioral activation was associated with more time at home for some, and the opposite for others. They point to the need for larger well-characterized longitudinal studies using innovative methods for annotating sensor data with behaviors or symptoms of interest in real-time.

Two papers provide foundational information around AI and affective computing. Renn et al. provide a concise primer on various clinically applicable forms of AI. They describe potential applications for diagnostics and treatment. Smith et al. focus on affective computing, defined as “study and development of systems and devices that can recognize, interpret, process, and simulate emotion.” The authors provide a detailed breakdown of clinical domains within depression and Alzheimer's disease and their quantification using markers generated by affective computing. The most consequential sections from both reviews highlight barriers and challenges to AI—namely the primarily theoretical potential of AI. While examples of the clinical impact of AI are emerging, the pace of AI tool development is tempered by concerns including absence of well-designed integration into clinical workflows and minimal cross-disciplinary training and infrastructure that is required for effective use of these tools.

One major concern shared across these reviews is the potential to build biased models based on non-representative samples. Older adults are at high-risk for exclusion from AI studies, due to decreased access and familiarity with technologies, though older adults have been shown to have capacity for learning and using tools with tailored programs (3). Datasets used to build AI algorithms must be representative of socioeconomic, regional, racial, and ethnic backgrounds to avoid building biased models with potentially negative clinical consequences. Equitable AI models will require targeted funding opportunities and an upfront focus on designing these algorithms to provide more equitable healthcare. Despite such challenges, the papers in this special issue provide insight and hope for AI tools to condense complex clinical data and incorporate novel data sources in the service of enhancing diagnostic and treatment approaches.

## Author Contributions

All authors contributed to conception of the editorial, wrote sections of the manuscript, contributed to manuscript revision, read, and approved the submitted version.

## Funding

HK was supported by the NIMH K01 Grant: K01MH122741. AI was supported by an Academic Scholars Award (Department of Psychiatry, University of Toronto) and the Walter and Maria Schroeder Institute for Brain Innovation and Recovery. IV was supported in parts by National Institute of Aging (NIA) Grants R01AG066670 and 3R01AG066670-02S1, The Once Upon a Time Foundation, the Eric Warren Goldman Charitable Fund of the National Philanthropic Trust and the McLean Hospital Institute for Technology in Psychiatry. EL was supported, in part, by the National Institute of Mental Health (NIMH) K23 Grant MH119375-01, the Stein Institute for Research on Aging at the University of California, San Diego, and the Veteran Affairs Healthcare System.

## Author Disclaimer

The content of this manuscript is solely the responsibility of the authors and does not necessarily represent the official views of the NIH.

## Conflict of Interest

The authors declare that the research was conducted in the absence of any commercial or financial relationships that could be construed as a potential conflict of interest.

## Publisher's Note

All claims expressed in this article are solely those of the authors and do not necessarily represent those of their affiliated organizations, or those of the publisher, the editors and the reviewers. Any product that may be evaluated in this article, or claim that may be made by its manufacturer, is not guaranteed or endorsed by the publisher.
